# Development of a MAGIC population and high-resolution quantitative trait mapping for nicotine content in tobacco

**DOI:** 10.3389/fpls.2022.1086950

**Published:** 2023-01-10

**Authors:** Guangdi Yuan, Kefan Sun, Wenlong Yu, Zipeng Jiang, Caihong Jiang, Dan Liu, Liuying Wen, Huan Si, Fengyan Wu, He Meng, Lirui Cheng, Aiguo Yang, Yuanying Wang

**Affiliations:** Key Laboratory of Tobacco Improvement and Biotechnology, Tobacco Research Institute, Chinese Academy of Agricultural Sciences, Qingdao, China

**Keywords:** tobacco, MAGIC population, GWAS, nicotine content, QTL

## Abstract

Multiparent Advanced Generation Inter-Cross (MAGIC) population is an ideal genetic and breeding material for quantitative trait locus (QTL) mapping and molecular breeding. In this study, a MAGIC population derived from eight tobacco parents was developed. Eight parents and 560 homozygous lines were genotyped by a 430K single-nucleotide polymorphism (SNP) chip assay and phenotyped for nicotine content under different conditions. Four QTLs associated with nicotine content were detected by genome-wide association mapping (GWAS), and one major QTL, named *qNIC7-1*, was mapped repeatedly under different conditions. Furthermore, by combining forward mapping, bioinformatics analysis and gene editing, we identified an ethylene response factor (ERF) transcription factor as a candidate gene underlying the major QTL *qNIC7-1* for nicotine content in tobacco. A presence/absence variation (PAV) at *qNIC7-1* confers changes in nicotine content. Overall, the large size of this MAGIC population, diverse genetic composition, balanced parental contributions and high levels of recombination all contribute to its value as a genetic and breeding resource. The application of the tobacco MAGIC population for QTL mapping and detecting rare allelic variation was demonstrated using nicotine content as a proof of principle.

## Introduction

Most traits of biological and economic interest in crop plants are under polygenic control, displaying complicated genetic structure and are extensively influenced by the interaction of genotype and environment (Falconer and Mackay, 1996; Doerge, 2002; Holland, 2007). Conventionally, the identification of genes underlying agronomic quantitative trait loci (QTLs) is performed using biparental mapping populations, such as recombinant inbred lines (RILs), and double haploid (DH) population (Rakshit et al., 2012). However, these populations have limitations due to the narrow genetic variation and limited opportunities for genetic recombination events, which lead to a lower map resolution (Valdar et al., 2006; Chen et al., 2013; Dell'Acqua et al., 2015; Huang et al., 2015).

Genome-wide association study (GWAS) provides another strategy for discovering genes and regions associated with agronomic traits using diverse population sets (Mitchell-Olds et al., 2010; Ongom and Ejeta, 2018). GWAS using SNP markers have made outstanding achievements in many economically important crops due to the exploitation of a large number of historical recombination events that lead to the rapid decay of linkage disequilibrium (Remington et al., 2001; Jaiswal et al., 2019). The mapping resources that have been widely employed for GWAS are accessions of landrace genetic resources or breeding lines that have been extensively phenotyped (Ongom and Ejeta, 2018). However, the main limitations of GWAS are linkage disequilibrium (LD), population substructure, and unbalanced allele frequencies (Mitchell-Olds, 2010; Visscher et al., 2012).

The main drawbacks of biparental populations and germplasm collections may be addressed by Multiparent Advanced Generation Inter-Cross (MAGIC) population (Mackay and Powell, 2007; Pascual et al., 2015; Scott et al., 2020). MAGIC population is a fine-scale mosaics of RILs that have roughly equal proportions of the founder genomes (Mackay and Powell, 2007; Cavanagh et al., 2008; Rakshit et al., 2012). The multiple founders of MAGIC population can enrich allelic diversity, whereas multiple intercrossing cycles result in a set of rearranged genomes with a high level of fragmentation, which gives greater opportunity for recombination and dramatically increases the power for QTL detection (Chen et al., 2013; Yamamoto et al., 2014; Arrones et al., 2020). Additionally, the restrictions of population structure and rare alleles for GWAS were mitigated to some extent in MAGIC population. Therefore, MAGIC population provides a compromise between the much greater complexity existed in naturally occurring accessions and the extreme simplicity of a diallelic system of RILs, making MAGIC an ideal material for GWAS analysis (Kover et al., 2009). To date, MAGIC population has been widely used in QTL mapping in many crop species. Bossa-Castro et al. (2018) identified 11 BSR QTLs effective against both bacterial leaf streak and bacterial blight and 3 pathovar-specific QTLs using an eight-way rice MAGIC population. Thyssen et al. (2019) identified genomic loci and candidate genes for six major fiber quality traits in a cotton MAGIC population using GWAS and whole genome sequencing. Rollar et al. (2021) used a Bavarian MAGIC wheat population for simple interval mapping and identified 19 QTLs corresponding to 11 distinct chromosomal regions controlling leaf rust resistance. However, to date, the construction and QTL mapping of MAGIC population have not been reported in tobacco.

Tobacco (*Nicotiana tabacum* L.) is an economically important crop and is also a model for the investigation of plant pathology, genetics, and biotechnology. Studies of tobacco genetics and genomics strengthened the use of *N. tabacum* as a plant model system and were also beneficial for tobacco molecular breeding. In tobacco, nicotine is an abundant predominant alkaloid produced exclusively in the roots and accumulating mainly in the leaves. It is derived from diamine putrescine, which depends on a series of metabolic enzymes, including putrescine N-methyltransferase (PMT), quinolinate phosphoribosyl transferase (QPT) and the PIP-family oxidoreductase A622 (Hibi et al., 1992; Sinclair et al., 2000; Kajikawa et al., 2009; Ryan et al., 2012). The biosynthesis of nicotine is genetically controlled by two distinct loci, NICOTINE1 (*NIC1*) and *NIC2* (Legg and Collins, 1971; Hibi et al., 1994). It has been reported that the clustered ethylene response factor (ERF) transcription factor at the *NIC2* locus can regulate nicotine content in tobacco by regulating PMT and QPT expression (Shoji et al., 2010; Qin et al., 2021). Sun et al. (2017) reported that nicotine content exhibited extreme variation in different tobacco accessions, and two significant SNP markers associated with nicotine content were identified in tobacco using the GWAS method. In this study, we developed the first eight-way MAGIC population in tobacco and identified genes/QTLs associated with nicotine contents using the MAGIC population. Based on 93,766 SNP markers and the phenotypic data obtained from three environments, four quantitative trait loci (QTLs) associated with nicotine content trait were mapped on Chr07 and Chr05 by GWAS. By combining genetic population research, bioinformatics analysis and gene editing, an ERF transcription factor was considered as the candidate gene underlying the major QTL *qNIC7-1* for nicotine content in tobacco. A presence/absence variation (PAV) at *qNIC7-1* confers changes in nicotine content. These results demonstrated the advantage and confidence of MAGIC population in the high-resolution detection of QTLs and genes responsible for complex traits.

## Materials and methods

### Tobacco MAGIC Population

**Figure 1:**
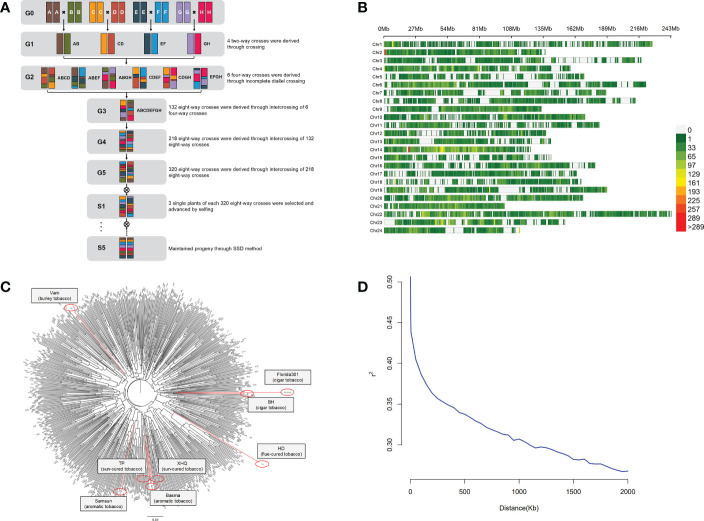
Diversity in the tobacco MAGIC population. **(A)** Breeding scheme of tobacco MAGIC population. Individual color represents each of eight parents (BH, Florida301, Vam, Basma, Samsun, XHQ, TP, HD). **(B)** Distribution of SNPs in 24 chromosomes.**(C)** Neighbor-joining tree of the 8 parents and 560 MAGIC lines, with parents labeled in red. **(D)** Decay of linkage-disequilibrium (LD).

A total of 8 tobacco accessions were used as parents of the MAGIC population. The construction of the MAGIC population used in this study is shown in **Figure 1A**. Beinhart1000-1 (BH) and Florida301 are cigar tobacco. Basma and Samsun are oriental tobacco. Xiaohuaqing (XHQ) and Tangpeng (TP) are sun-cured tobacco from China. Vam belongs to burley tobacco. Honghuadajinyuan (HD) is a flue-cured tobacco cultivar. The first stage was pairwise crossing of eight parents, and 4 two-way crosses were produced. The resulting 4 two-way crosses were incomplete diallel crossed to produce 6 four-way crosses. The 6 crosses were then intercrossed to produce 132 eight-way crosses. To increase the number of recombination events, the resulting eight-way crosses were intercrossed to produce 218 eight-way crosses, and the 218 crosses were randomly mated and produced 320 eight-way crosses. Three single plants of each 320 eight-way crosses were selected and propagated by single seed descent (SSD) method during five selfing generations. Finally, more than 800 eight-way tobacco MAGIC homozygous lines were constructed.

### Phenotypic data analysis

Five hundred and sixty MAGIC homozygous lines (MLs) and 8 parents were planted in Zhucheng, Shandong, China (119.40_E, 36.99_N, 61 m altitude), in 2019 and 2020 (ZC2019 and ZC2020) and in Chenzhou, Hunan, China (113.01_E, 25.78_N, 190 m altitude), in 2020 (CZ2020) with a randomized complete block design with two replications. In all trials, one replication of each line contained two 10-plant rows with a row length of 10 m, row spacing of 1.2 m and plant distance of 0.5 m. Two weeks after topping, two mature middle leaves from each plant were harvested and mixed as one sample. Three plants of each line were randomly sampled for nicotine content measurement, and the average value of three plants was used for further analysis. Nicotine content was measured using a Continuous Flow Analyzer (SAN++, Skalar Analytical B.V., Breda, the Netherlands) from ground cured leaves. Variance analysis was performed using QTL Icimapping version 4.2.53.

### Genotyping by SNP array

A total of 560 MAGIC lines and 8 parents was genotyped using the 430K tobacco SNP array with 432,362 markers at Zhengzhou Tobacco Research Institute of CNTC. Details of the genotyping and SNP calling procedures are described by Zhang et al. (2017). SNPs with no polymorphism among 8 parents, missing rate > 10% and minor allele frequency (MAF) < 0.05 were removed. Finally, a total of 93,766 SNP markers were used for subsequent studies (**Table S1**).

### Linkage disequilibrium determination

The genome-wide LD between pairs of loci was performed using Tassel 5.0 software (Bradbury et al., 2007). The estimates of the LD were measured using the squared allele-frequency correlations (r^2^) for pairs of loci. The distance in base pairs that loci could be expected to be in LD or LD decay was computed by plotting r^2^ onto physical distance using the threshold r^2 =^ 0.33 (one-third of the maximum value) as a cutoff. The LD contour plot was generated from PopLDdecay software (Zhang et al., 2019).

### Genome-wide association study

All marker-trait associations were performed using TASSEL 5.0 software. A mixed linear model (MLM) was employed for GWAS analysis by incorporating a kinship matrix along with PCA. SNPs with minor allele frequency (MAF) <0.05, no polymorphisms and deletion rates higher than 10.0% were filtered out. Components of PCA were set as 5. Pedigree analysis (K) kinship method chose Centered_IBS with Max Alleles as 6. The compression level of the MLM is the optimum level, and P3D is selected for variance component estimation. Manhattan and Q-Q plots were derived from the GWAS results using the CMplot package (https://github.com/YinLiLin/R-CMplot) within R software (http://www.r-project.org/). For results with inflation, genomic control was applied to further control the confounding with population structure (Yurii et al., 2007). The Bonferroni correction method based on the FWER (Family-Wise Error Rate) standard was used to assess the significance threshold of association sites, and the significance level was determined as *p* = 1.0E-07 (0.01/93,766). When multiple loci were associated with a trait and were within a 3 Mb interval, they were considered as a single QTL.

### Resequencing and data analysis

Four MAGIC parent cultivars (BH, HD, Florida301, Samsun) and one flue-cured tobacco cultivar (XHJ) were used for resequencing. Genomic DNA of the 5 tobacco accessions was randomly fragmented to 350 bp for library construction. The constructed library was sequenced on the Illumina NovaSeq 6000 platform by Novogene, Beijing, China, and 150 bp paired-end reads were generated. Furthermore, quality-controlled data were generated after removing reads with ≥10% unidentified nucleotides (N); >10 nt aligned to the adapter, allowing ≤10% mismatches; >50% bases having Phred quality <5 and putative PCR duplicates generated in the library construction process. After quality control of the raw data, the clean reads were mapped to the reference genome.

The candidate regions (83 Mb - 86 Mb) of chromosome 7 from five sequenced cultivars were compared, and the average coverage depth was counted using Tablet software (James Hutton Institute) with a window size of 100 kb.

### DNA extraction and candidate gene cloning

Total DNA was extracted from fresh tobacco leaves using a CTAB method. The quality and quantity of total DNA was determined using a NanoDrop 2000 spectrophotometer (NanoDrop Technologies Inc., Wilmington, DE, USA). PCRs were performed with 2× Phanta Flash Master Mix (Dye Plus) and gene-specific primers according to the manufacturer’s instructions. PCR products were analyzed by electrophoresis and purified using FastPure Gel DNA Extraction Mini Kit (Vazyme). The purified DNA fragment was ligated into the *pEASY*-T1 vector (Transgen) and then transformed into *Escherichia coli* Trans5a competent cells (Transgen) for sequencing. All primer information is listed in **Table S2**.

### Generation of CRISPR/Cas9-mediated mutants

CRISPR/Cas9-mediated target mutagenesis was used to knock out the candidate genes, *qNIC7-1* (*NtERF170*, *NtERF199*, and *NtERF890*) and *NtERF189*, the homologous gene of *NtERF199*. The targeting sequences (listed in **Table S2**) and their reverse complement oligomer were chemically synthesized and annealed, and the dimers obtained were then inserted into the PHSE401 binary vector. For multigene editing, the dimers are connected with the tRNA sequence.

The constructs obtained above were individually transformed into competent *Agrobacterium tumefaciens* strain EHA105 cells using the freeze–thaw method. Leaf discs from 6-week-old XHJ were infected with *A. tumefaciens* strain EHA105 harboring the knockout vectors following the methods reported (Wang et al., 2022). The infected leaf discs were then plated onto generation medium (MS medium containing 3% (w/v) sucrose, 1 mg/L 6-benzylaminopurine, 0.15 mg/L 1-naphthaleneacetic acid, 50 mg/L cefotaxime sodium, and 8 mg/L hygromycin, pH 5.7). Hygromycin-resistant seedlings were obtained, and genomic DNA was extracted. *NtERF170*, *NtERF189*, *NtERF199* and *NtERF890* were amplified by PCR from genomic DNA, and the PCR products were Sanger sequenced to detect the mutations.

### Screening of homozygous mutants and nicotine content measurement

Single-gene (M170, M199, M890) and multigene (M-2, M-3) T2 generation knockout mutants were obtained, and homozygous screening was conducted. The DNA of each individual plant was extracted. Full-length primers were used to amplify target genes, and the PCR products were purified and ligated into *pEASY*-T1 vectors for sequencing. At least ten clones from each sample were sequenced. Only when all clones had identical mutations were the corresponding plants considered as homozygous mutants.

Mature middle leaves of 12-week-old plants were collected, dried in an electro-thermostatic blast oven, and ground using a tissue grinder (Jingxin Industrial Development Co., Ltd., Shanghai, China). Fifty milligrams of ground leaves were sampled for nicotine content measurement on a HITACHI Chromaster (autosampler Chromaster 5210, column oven Chromaster 5310, diode array detector Chromaster 5430, RI detector Chromaster 5450). Leaves collected from three independent plants and mixed together were considered as one replicate. Three replicates were measured for each mutant line.

### Distribution analysis in germplasm resources

Different accessions were obtained from the National Medium-term GenBank of Tobacco Germplasm Resources of China. A total of 1,187 flue-cured tobacco accessions and 114 cigar accessions were planted in the field in Jimo, Shandong, China (120°44’71’’E, 36°38’93’’N, 22 m altitude) in 2020. The nicotine content was measured as described above. Leaf samples were collected, and genomic DNA was extracted. *NtERF189* and *NtERF199* were amplified in 1301 DNA samples using both full-length primers and short segment primers. PCR products were analyzed by electrophoresis, and those with no target amplification were considered as target gene deletions.

## Results

### Tobacco MAGIC population structure assessment

Eight tobacco accessions (BH, Florida301, Vam, Basma, Samsun, XHQ, TP, HD) that belong to different tobacco types were used to produce the MAGIC population (**Figure 1A**). The effectiveness of random mating and its consequence on the structure of this population was evaluated using 93,766 SNP markers distributed on 24 chromosomes (**Figure 1B**). Population structure analysis by the neighbor-joining method indicated that no specific population structure exists among the 560 MAGIC lines (**Figure 1C**). Kinship analysis also showed no specific clustering pattern (**Supplementary Figure 1**). To determine the mapping resolution for GWAS, the average extent of LD decay was quantified (**Figure 1D**). Using the whole set of SNPs, the LD decay rate of the population for the entire genome was estimated to be 1,500 kb, with r^2 =^ 0.33 (one-third of the maximum value). All data indicated that the tobacco MAGIC population was suitable for GWAS analysis with no obvious population structure and moderate LD level.

### Phenotypic and genotypic evaluation of the MAGIC population

**Figure 2:**
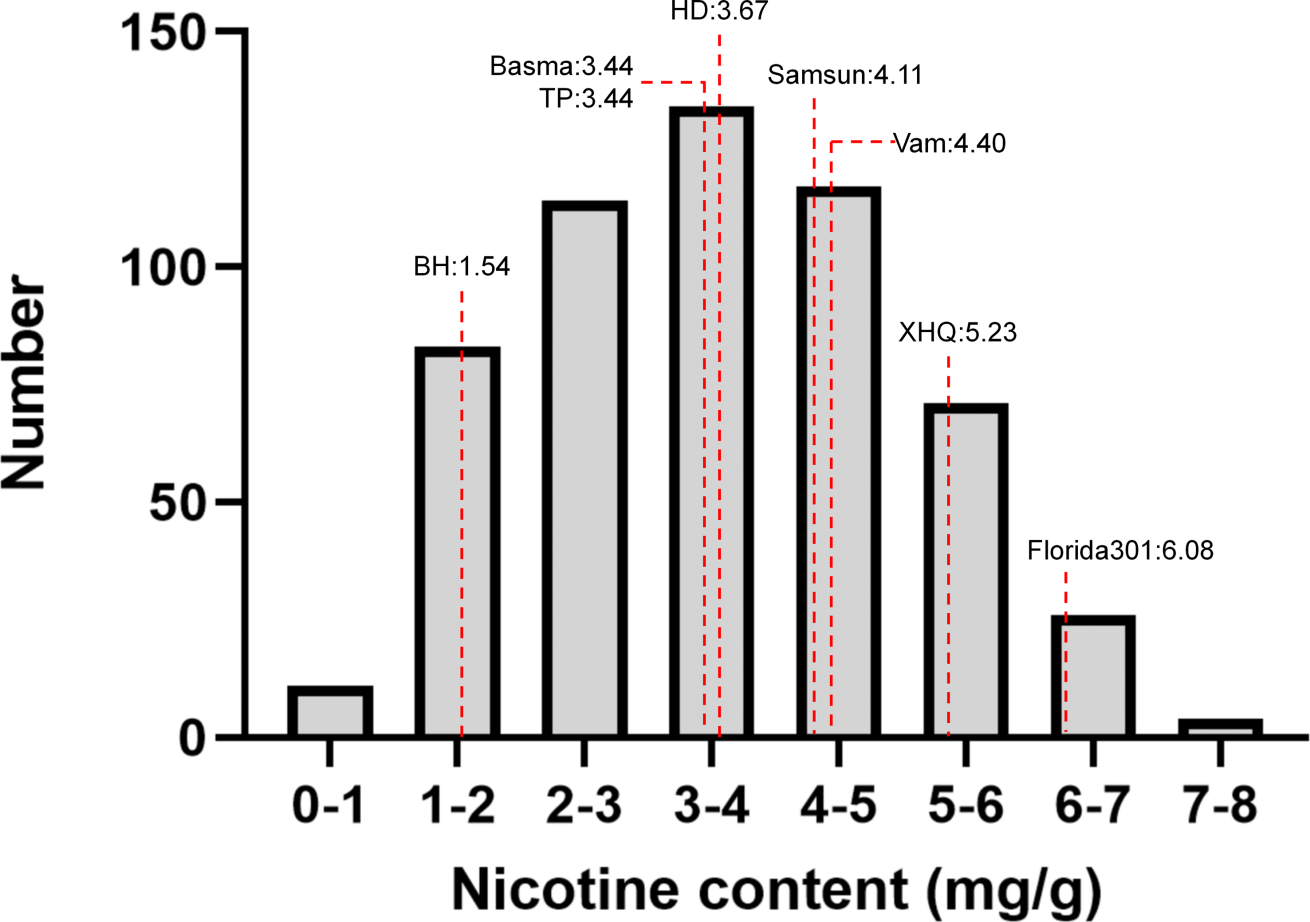
Phenotypic distribution of 8 parents and 560 MAGIC lines. Eight parents were shown as dotted lines.

Nicotine content data of 8 parents and 560 MLs were collected from three environments (2019 in Zhucheng, 2020 in Zhucheng, 2020 in Chenzhou). In this study, the nicotine content of mature middle leaves was measured, and the results are shown in **Figure 2** and **Supplementary Table 3**. As shown in **Table 1**, the mean values of parents and 560 MLs were similar. However, the range of variation was more extensive in MLs, possibly due to transgressive segregation. The variation in nicotine content was determined by genotype and environment. The heritability was approximately 0.5 by plot, demonstrating that the trait was quantitatively inherited.

**Table 1:** Phenotypic variations, variance components and heritability for nicotine content in tobacco MAGIC population in 3 environments.

	Range of Variation(mg/g)	Mean^a^(mg/g)	SD^b^	Mean squares				Heritability (%)	
				Environment	Genotype	G×E	Error	Plot level	Genotypic mean level
Parents	1.54-6.08	3.99	1.35	15.26	6.94	2.16	0.41	46.27	75.18
MLs	0.34-7.55	3.56	1.45	922.10	11.88	2.40	0.77	54.62	82.21

a Average nicotine content.

b Standard deviation.

### Genome-wide association analysis of nicotine content

**Figure 3:**
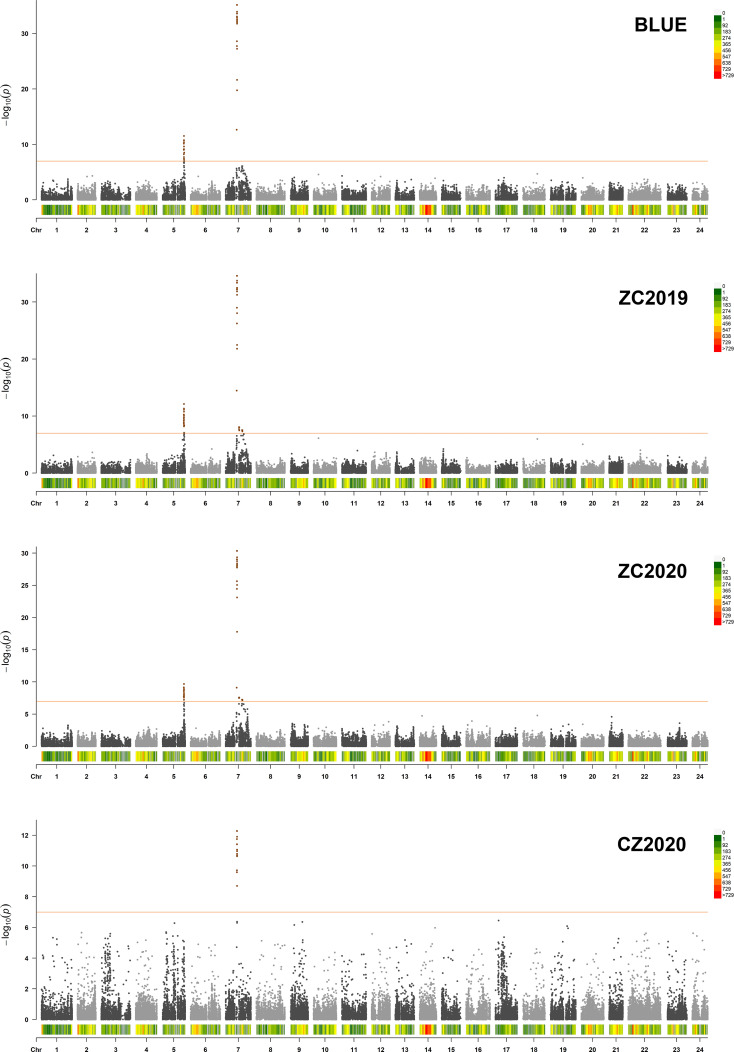
Manhattan Plot for nicotine content generated from GWAS analysis following mixed linear model (MLM). X-axis and Y-axis represent physical position of 24 chromosomes and negative log_10_(*p*-value), respectively. Different colors at the top right corner and bottom of manhattan plot represent different densities of SNP markers. Horizontal solid line represents the significant threshold (*p* =1.0E-07). SNP markers with *p <*1.0E-07 were colored in dark orange.

GWAS was carried out using the best linear unbiased estimates (BLUEs) of MLs over three replicates in a mixed linear model (MLM) by employing Tassel 5.0 software. The quantile-quantile (Q-Q) plot showed that most of the observed *p* values followed a uniform distribution, but the few that were in LD with a causal polymorphism had significant *p* values in the tail (**Supplementary Figure 2**). The Manhattan plot for nicotine content was generated from TASSEL software and the R package “CMplot” (**Figure 3**). At genome wide significance (*p* value ≤ 1.0E-07), a total of 4 QTLs (*qNIC7-1, qNIC7-2, qNIC7-3, qNIC5-1*) distributed on chromosome 7 and chromosome 5 were identified (**Table 2**). Among them, *qNIC7-1* and *qNIC5-1* were repeatedly detected in most environments. The other 2 QTLs (*qNIC7-2*, *qNIC7-3*) were only identified in ZC2019 and ZC2020.

**Table 2:** Summary of QTLs associated with nicotine content in tobacco across different environments.

QTL	SNP	Position	ZC2019^a^		ZC2020^a^		CZ2020^a^		BLUE^b^	
			*p*	effect	*p*	effect	*p*	effect	*p*	effect
*qNIC7-1*	AX-117513847	84,056,268	2.59E-35	2.57	4.49E-31	1.90	5.21E-13	1.97	6.85E-36	2.09
	AX-117513846	84,098,896	2.59E-35	2.57	4.49E-31	1.90	5.21E-13	1.97	6.85E-36	2.09
	AX-117632223	84,282,622	2.59E-35	2.57	4.49E-31	1.90	5.21E-13	1.97	6.85E-36	2.09
	AX-117522763	84,740,457	2.59E-35	2.57	4.49E-31	1.90	5.21E-13	1.97	6.85E-36	2.09
*qNIC7-2*	AX-117702600	99,815,696	8.95E-09	-0.05	2.66E-08	-0.27	/	/	/	/
*qNIC7-3*	AX-117503751	121,872,699	2.85E-08	1.54	5.77E-08	1.16	/	/	/	/
	AX-117538002	124,443,130	2.85E-08	1.54	5.77E-08	1.16	/	/	/	/
*qNIC5-1*	AX-117702271	160,770,794	4.82E-12	1.16	2.10E-10	0.77	/	/	3.01E-12	0.92

a ZC2019, ZC2020 and CZ2020 refer to Zhucheng in 2019, Zhucheng in 2020 and Chenzhou in 2020.

b The best linear unbiased estimates

The most significant locus, *qNIC7-1*, associated with nicotine content had 19 markers spanning 772 kb (**Supplementary Table 4**). The SNPs with the strongest association (AX-117513847, AX-117513846, AX-117632223, AX-117522763, *p* = 6.85E-36) explained 32.6% of the phenotypic variation. Based on the results of LD decay analysis and association analysis, we estimated the candidate region of *qNIC7-1* to be 83 Mb - 86 Mb, which contained 28 SNPs and 41 annotated genes (**Supplementary Table 5**). In *qNIC5-1*, the SNP with the strongest association (AX-117702271, *p* = 3.01E-12) explained 9.2% of the phenotypic variation. The candidate region of *qNIC5-1* was estimated to be 159.5 Mb-162.5 Mb, which contained 64 SNPs and 46 annotated genes (**Supplementary Table 5**).

### Candidate gene analysis

**Figure 4:**
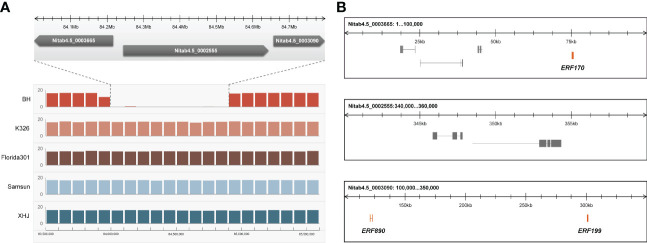
Candidate gene analysis. **(A)** Resequencing data of 5 tobacco accessions (BH, K326, Florida301, Samsun, XHJ) revealed a fragment deletion in BH. X-axis represent position of Chr07 with the window size of 100kb. Y-axis represent average coverage depth of resequencing data. The deletion fragment was mapped to 3 scaffolds (Nitab4.5_0003665, Nitab4.5_0002555, Nitab4.5_0003090) of K326. **(B)** The 3 scaffolds of K326 corresponding to the deleted fragment comprises of three ERFs (*NtERF170*, *NtERF199* and NtERF870).

**Figure 5:**
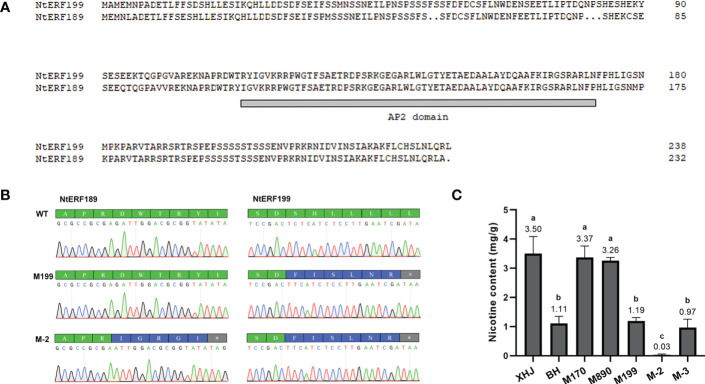
Validation of the association between two ERFs and nicotine content. **(A)** Alignment of *NtERF199* and *NtERF189*. The AP2 domain is indicated by a gray line. **(B)** Mutation of *NtERF189* in M-2 and *NtERF199* in M199 and M-2. Normal amino acids are marked in green. Mutated amino acids are marked in blue and stop codon is marked in grey. **(C)** Nicotine content of wildtype (XHJ, BH) and T2 homozygous mutants (M170, M890, M199, M-2, M-3).

We noticed that eight tandem ERF transcription factors were located on the region covering the strongest-associated SNPs (84 Mb - 85 Mb) in *qNIC7-1*. Resequencing data analysis showed that three of the ERFs (*NtERF170*, *NtERF199*, and *NtERF890*) were deleted in one parent cultivar, Beinhart1000-1, which had the lowest nicotine content, whereas the remaining seven showed no sequence or expression differences (**Figure 4**). Therefore, we focused on the three deleted ERFs. To examine whether their deletions are responsible for nicotine content reduction, we used CRISPR/Cas9 technology to generate loss-of-function mutants of three genes separately (M170, M199, M890) and collectively (M-3) in the flue-cured tobacco cultivar XHJ and evaluated their phenotypic effect in the T2 generation. The genotypes of wild-type (XHJ), single-gene homozygous mutants (M170, M199, M890) and multigene homozygous mutant (M-3) plants were determined by DNA sequencing. The average nicotine content of XHJ was 3.50 mg/g. The average nicotine contents of M170 and M890 were 3.37 mg/g and 3.26 mg/g, respectively, showing no obvious differences compared to the wild type. However, M199 and M-3 exhibited significant decreases with the nicotine contents of 1.19 and 0.97 mg/g, approximately 1/3 of that in wild-type XHJ, corresponding to the nicotine level in BH (1.11 mg/g) (**Figure 5**).

BLAST analysis showed that *NtERF189* on chromosome 19 is homologous to *NtERF199*, sharing an identical binding domain. To investigate the effect of both genes on nicotine synthesis, we also generated a two-gene mutant (M-2) in which *NtERF189* and *NtERF199* were collectively knocked out (**Figure 5**). As expected, nicotine was barely detectable in M-2 plants (0.03 mg/g), approximately 1/100 of that in WT plants, 1/30 of BH, M199 and M-3.

The candidate region of *qNIC5-1* contained 46 annotated genes. According to the annotation and gene function analysis, we predicted two candidate genes (*NIC5-1* and *NIC5-2*) that might be associated with nicotine content. *NIC5-1* was annotated as a MYB35 transcription factor and located 304 kb downstream of AX-117702271. *NIC5-2* was located 1,188 kb downstream of AX-117702271 and homologous to the MYC2 transcription factor. The functions of the two genes in nicotine synthesis need to be further studied in the future.

### Distribution analysis of mutations in germplasm resources

**Figure 6:**
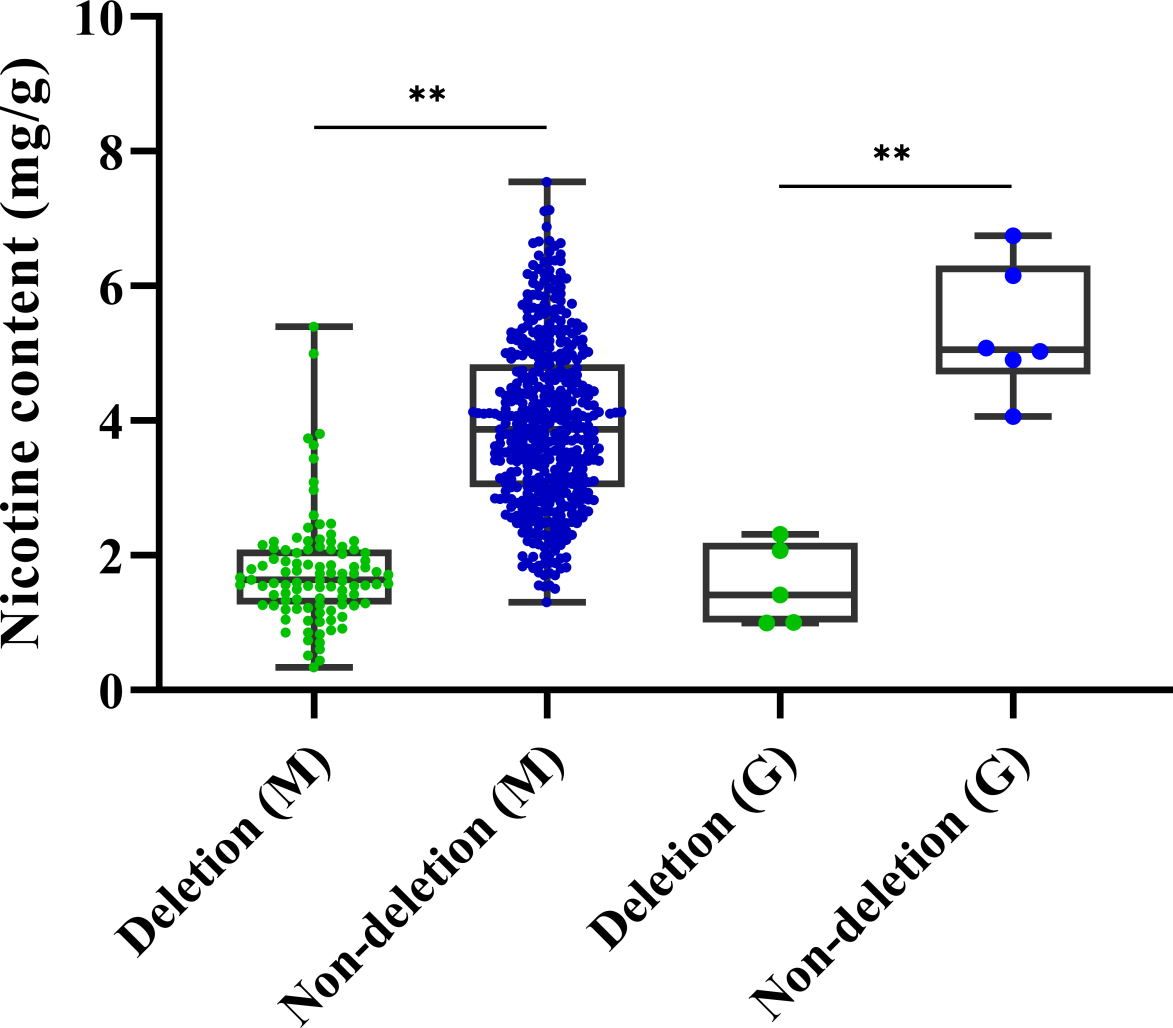
Boxplots for nicotine content of deletion type and non-deletion type in 560 MAGIC lines and germplasm resources. Significance level was calculated by one-way ANOVA (**P < 0.01, *P< 0.05).

To explore the practical utility of the deletion mutation in *qNIC7-1*, we designed an insertion-deletion (InDel) marker (NIC7001) (forward primer: 5’-GATGTGGGTGTCAACCTTTTCG-3’ and reverse primer: 5’-CGAACCCTTCCTTTCACATAA-3’) and genotyped 560 MLs and 1301 tobacco accessions. The InDel marker NIC7001 locus has two types: deletion type and nondeletion type (839 bp amplification). The two types were compared using one-way ANOVA using IBM SPSS Statistics software version 23 (IBM, New York, USA). In the MAGIC population, the lines of the nondeletion type had a mean nicotine content of 3.95 mg/g, which was significantly higher than the mean nicotine content of the deletion type lines (1.77 mg/g). In 1301 tobacco cultivars, which included 1187 flue-cured tobacco accessions and 114 cigar accessions, only 11 cultivars (8 flue-cured tobacco types and 3 cigar tobacco types) were detected as deletion types (**Table 3**). Five out of the 11 cultivars, together with six nondeletion-type cultivars, were phenotyped. In accordance with the results in MAGIC lines, the nicotine content of deletion type cultivars was significantly lower than that of nondeletion type cultivars (**Figure 6**).

**Table 3:** Distribution of deletion mutation of *NtERF189* and *NtERF199* in germplasm resources.

	Accessions Tested	*NtERF199* deleted	*NtERF189* deleted	Both deleted
Flue-cured tobacco accessions	1187	8 (0.67%)	14 (1.18%)	0
Cigar accessions	114	3 (2.63%)	15 (13.16%)	0
Total	1301	11 (0.85%)	29 (2.23%)	0

The distribution of *NtERF189* (NIC2) deletion was also investigated using the InDel marker NIC7002 (forward primer: 5’-GCGTGCAATAGGGCAAAGC-3’ and reverse primer: 5’-TGAAAGCTTCCTTCCTTTCACA-3’) in 1301 tobacco germplasm cultivars (**Table 3**). The deletion of *NtERF189* was detected in 14 flue-cured tobacco accessions (1.18%) and 15 cigar accessions (13.16%), with a total deletion rate of 2.23%. *NtERF199* was deleted in only 8 flue-cured tobacco accessions (0.67%) and 3 cigar accessions (2.63%), with a total deletion rate of 0.85%. No accession was identified that carried deletions in both genes.

## Discussion

### The MAGIC population exhibits more power and potential for QTL mapping

To identify genetic variation controlling complex traits variation, many biparental populations have been widely used for QTL mapping in tobacco (Drake-Stowe et al., 2017; Sun et al., 2018; Agacka-Mołdoch et al., 2021). However, the main limitations of biparental population for QTL mapping are the low genetic diversity of the mapping population and mapping resolution. In MAGIC population, multiple founders are intercrossed several times in a well-defined order to combine the genetic material of all the founders in a single line (Cavanagh et al., 2008). Therefore, compared to biparental mapping population, MAGIC population contains more recombination events and genetic diversity, which enhances mapping resolution and the ability to analyze several alleles simultaneously. In this study, eight founders in the MAGIC population enriched population with higher allelic diversity compared to those derived from the traditional biparental populations. Furthermore, we narrowed down the target region, and *NtERF199* was considered exactly as the candidate gene by MAGIC population. In addition, the distribution of *NtERF199* in tobacco germplasm resources was analyzed. The deletion of *NtERF199* was considered as a rare allelic variation (RAV) with a deletion rate of 0.85%. Sun et al. (2017) performed GWAS using 219 flue-cured tobacco accessions and identified 2 QTLs associated with nicotine content by GWAS. However, the most major-effect QTL (*qNIC7-1*) in this study was not identified using the 219 flue-cured tobacco accessions, probably due to the filteration of RAV. Because the crossover technique elevates the frequency of all parental alleles to be almost equal, MAGIC population have higher power to identify low frequency alleles and can better evaluate allelic effects between founders than genome-wide association panels (Dell'Acqua et al., 2015; Scott et al., 2020). Another main advantage of MAGIC population over association panels is the lack of an underlying unknown structure, which enhances the risk of detecting false-positives. Therefore, MAGIC population represents an intermediate resources between biparental crosses and association panels regarding the number of traits that can be investigated, resolution, allele diversity and population structure (Rakshit et al., 2012; Pascual et al., 2016). We believe that this tobacco MAGIC population provides a good resource for studying the genetic basis of complex traits in tobacco.

### GWAS and identification of candidate genes for nicotine content

GWAS is a forward genetic approach to identify underlying causal genes, mutations and putative functional markers that affect complex quantitative traits. In this study, we identified four QTLs distributed on two chromosomes associated with nicotine content. The candidate gene *NtERF199* on *qNIC7-1* and its homologous gene *NtERF189* were cloned and experimentally verified through loss-of-function mutant analysis. The results are consistent with previously reported studies. Shoji et al. (2010) identified that the *NIC2* locus comprises a cluster of ERFs (including *NtERF189*) that regulate nicotine biosynthesis in tobacco. Qin et al., (2021) validated *NtERF199* as the *NIC1* gene. The detection of *qNIC7-1* highlighted the strength of using tobacco MAGIC population in GWAS.

As in the case of *NIC2* locus in tobacco, eight ERFs are clustered together in the *qNIC7-1* locus. However, both the previous study by Qin et al., (2021) and our investigation confirmed that *NtERF199* is the only causal gene for nicotine biosynthesis within the *qNIC7-1* locus. Same case also exists in tomatoes, with *GAME9* locates within an ERF cluster and works as the only functional regulator of steroidal glycoalkaloid biosynthesis (Cardenas et al., 2016). These results suggest potential functional redundancy and divergence of clustered ERFs.

Allopolyploid plants usually display higher adaptability than their diploids ancestors since their preexisting homeologous loci from the progenitors show a predominantly additive gene expression pattern (Fu et al., 2016). As a natural allopolyploid plant, *N. tabacum* was formed by hybridization of *Nicotiana sylvestris* (S-genome) and *Nicotiana tomentosiformis* (T-genome) (Clarkson et al., 2017). Blast analysis and functional studies have demonstrated that *NtERF199* and *NtERF189* are a pair of homologous genes originated from *N. sylvestris* and *N. tomentosiformis*, respectively, and showed dose-dependent effects on nicotine levels (Shoji et al., 2010; Qin et al., 2021). It has also been proven through transcriptome analysis that most homologous genes from the S- and T-genomes of tobacco have been maintained and are expressed in a similar way (Bombarely et al., 2012; Edwards et al., 2017). Therefore, the effect of homologous genes must be taken into consideration in the study of gene function in allotetraploid plants, especially *N. tabacum*.

We also identified a significant QTL on chromosome 5 (*qNIC5-1*), which explained 9.6% of the phenotypic variation. Among the 46 annotated genes in *qNIC5-1*, we predicted two candidate genes (*NIC5-1* and *NIC5-2*) likely associated with nicotine content based on the annotation and gene function analysis. *NIC5-1* was annotated as a MYB35 transcription factor. MYB35 has been reported to regulate methyl jasmonate (MeJA) and wound-responsive G10H-1 in *Bacopa monnieri* (Jeena et al., 2021). Nicotine biosynthesis in tobacco is highly regulated by jasmonic acid (JA) (Baldwin et al., 1996; Shoji et al., 2008). Therefore, MYB35 might participate in nicotine synthesis by regulating JA-responsive and nicotine biosynthesis-associated genes. *NIC5-2* was annotated as a MYC2 transcription factor, which has been described to control nicotine biosynthesis genes by directly binding the G-box in the target promoters and upregulating the *NIC2*-locus ERF genes (Shoji and Hashimoto, 2011; Zhang et al., 2012; Sui et al., 2021).

### Development of functional markers and distribution of allelic variation

To investigate the distribution of *NtERF189* and *NtERF199* deletion, we manually designed two InDel markers (NIC7001 and NIC7002) and screened a total of 1301 cultivated tobacco accessions, including 1187 flue-cured tobacco accessions and 114 cigar tobacco accessions. In flue-cured tobacco, the deletion mutation rates of *NtERF189* and *NtERF199* were 1.18% and 0.67%, respectively, while in cigar tobacco, they reached 13.16% and 2.63%, respectively. The results demonstrated that the two ERF deletion mutations displayed higher frequency in cigar tobacco. In fact, the low-nicotine trait was initially found in strains of Cuban cigar tobacco varieties and subsequently introduced into cigarette varieties through a series of backcrosses (Valleau, 1949). This might explain the higher mutation frequency in cigar tobacco. Overall, the deletion mutation of *NtERF189* and *NtERF199* was detected in only 2.23% and 0.85% of the tested tobacco cultivar, demonstrating them as rare allelic variations. Deletion of both genes was detected in no accessions. This may be attributed to the importance of nicotine in resisting biological stresses, and those with both ERFs mutated have already been naturally eliminated.

Moreover, we validated the effectiveness of NIC7001 using a panel of 11 tobacco cultivars (5 deletion types and 6 nondeletion types). The results provide promising evidence of InDel marker application for future breeding programs of low nicotine tobacco cultivars.

## Conclusion

This work reports the first MAGIC population in tobacco, constructed from eight common breeding lines. By using the MAGIC population with negligible population structure coupled with high-density SNP markers, GWAS was performed and identified four QTLs associated with the low nicotine trait. The candidate gene *NtERF199* in *qNIC7-1* and its homologous gene *NtERF189* were analyzed and verified, which proved the reliability of the GWAS results in the MAGIC population. Candidate genes in *qNIC5-1* were also analyzed and need to be further studied. Overall, the tobacco MAGIC population provides for the identification of QTLs associated with other complicated important traits.

## Data availability statement

The datasets presented in this study can be found in online repositories. The name of the repository and accession number(s) can be found below: NCBI Sequence Reads Archive (SRA) database (Accession number: PRJNA904563).

## Author contributions

GY, KS, WY, ZJ, LW, and FW performed the experiment. GY, LC and HS analyzed the experimental data. CJ, DL and HM participated in the field trails. YW, AY and LC designed the experiment. GY and LC wrote this manuscript. All authors contributed to the article and approved the submitted version.

## Supplementary material

Supplementary Figure 1 Kinship matrix among the 560 MAGIC lines of tobacco MAGIC population using SNP markers.

Supplementary Figure 2 Q-Q plots of nicotine content across different environments.
