# Supplementary figures and images for: Development of a MAGIC population and high-resolution quantitative trait mapping for nicotine content in tobacco
Source: Front Plant Sci. 2023 Jan 10;13:1086950. doi: 10.3389/fpls.2022.1086950 (PMC9871594; doi:10.3389/fpls.2022.1086950)

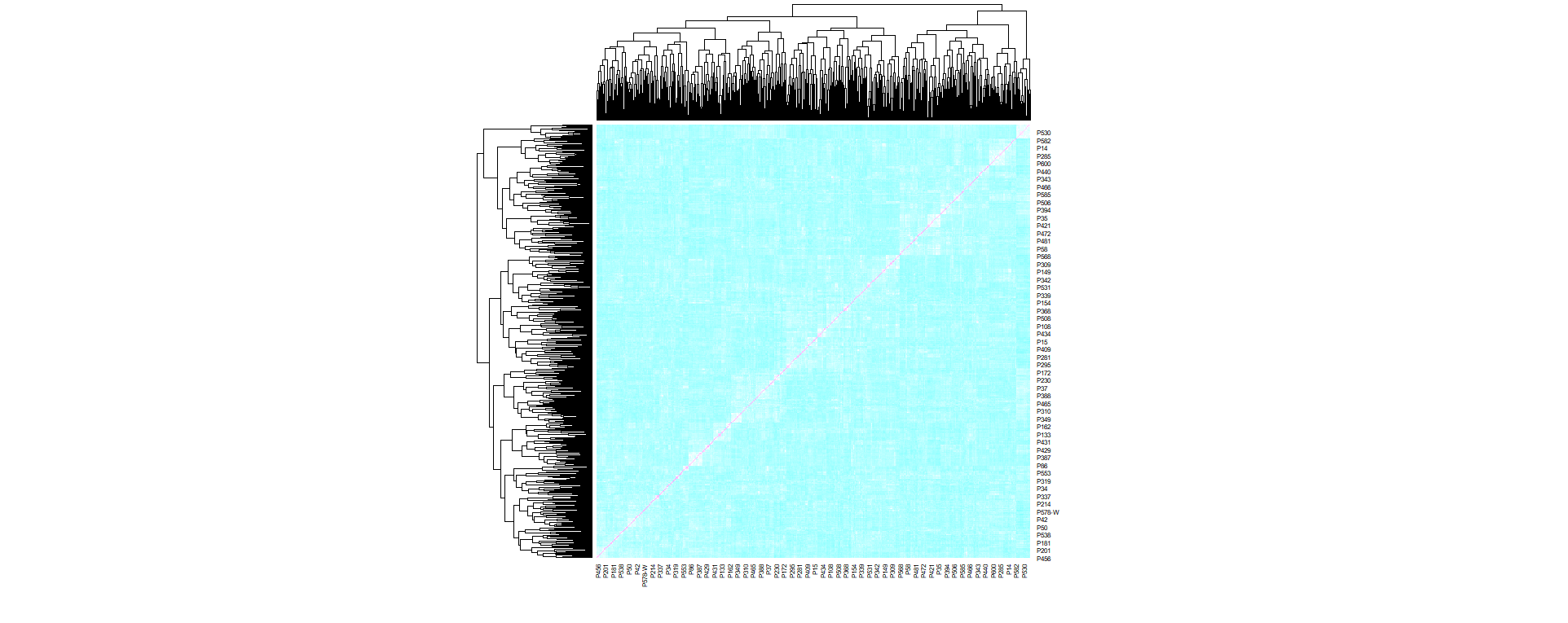

Supplement: Supplementary Figure 1 — Kinship matrix among the 560 MAGIC lines of tobacco MAGIC population using SNP markers. [file DataSheet_1.zip › Image 1.TIFF]

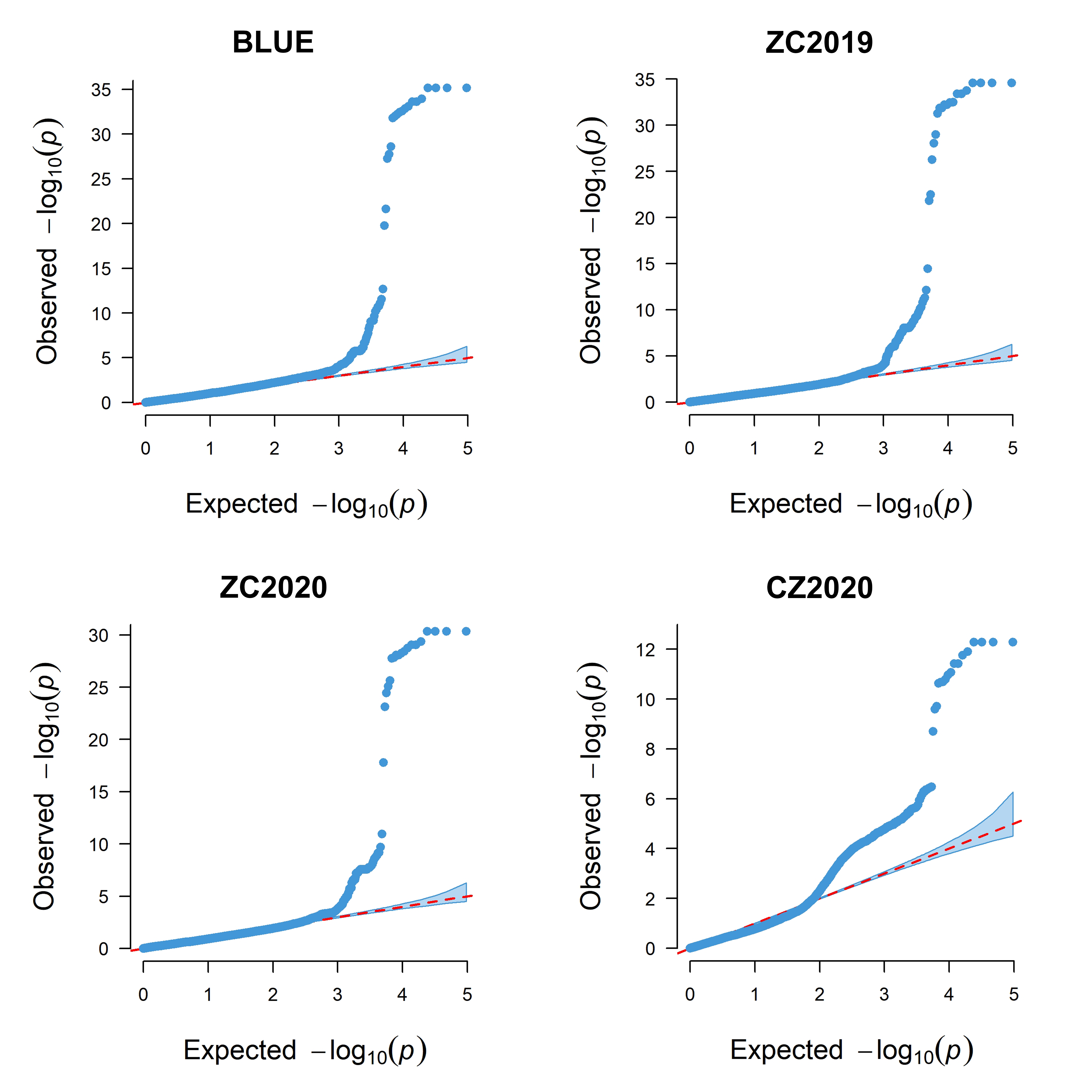

Supplement: Supplementary Figure 1 — Kinship matrix among the 560 MAGIC lines of tobacco MAGIC population using SNP markers. [file DataSheet_1.zip › Image 2.png]
